# Profoundly Delayed Emergence due to Pulmonary Embolism

**DOI:** 10.1155/cria/6691273

**Published:** 2025-02-18

**Authors:** Allison Steinauer, Whitney Marvin, Natalie R. Barnett

**Affiliations:** ^1^Department of Pediatrics, University of North Carolina Health, Chapel Hill, North Carolina, USA; ^2^Department of Pediatrics, Medical University of South Carolina, Charleston, South Carolina, USA; ^3^Department of Anesthesia and Perioperative Medicine, Medical University of South Carolina, Charleston, South Carolina, USA

**Keywords:** case report, delayed emergence from anesthesia, intensive care units, pediatric, pediatric obesity, pulmonary embolism

## Abstract

Pulmonary embolism is a rare, but serious, potential perioperative complication and crisis. We present a case of a 12-year-old female undergoing distal femoral and proximal tibial osteotomies with internal fixations who experienced acute onset hypercapnia, tachycardia, and prolonged minimal responsiveness following deflation of the tourniquet and cessation of inhaled sevoflurane. CT-chest demonstrated bilateral partially occlusive filling defects of the pulmonary vasculature. We concluded that the patient experienced a pulmonary embolism resulting in V/Q mismatch, retained sevoflurane, and ultimately delayed emergence.

## 1. Introduction

Pulmonary embolism (PE) is a potentially catastrophic perioperative event and is fortunately uncommon in pediatrics. Approximately 95% result from embolization of deep vein thrombosis (DVT) [1]. Common symptoms include tachycardia, hypoxemia, hypotension, and decreased end-tidal carbon dioxide (EtCO_2_) [1]. We report a case of delayed emergence in a pediatric patient likely secondary to embolism-induced ventilation/perfusion (V/Q) mismatch. This case report was prepared in accordance to the CARE checklist for case reports (Supporting Document 1).

## 2. Case Presentation

A 12-year-old Black female with morbid obesity (130.5 kg, BMI 47.36 kg/m^2^), well controlled asthma, obstructive sleep apnea, and leg length discrepancy and pain secondary to genu valgum presented for distal femoral and proximal tibial osteotomies with internal fixation. She was premedicated with midazolam 2 mg intravenously. General anesthesia was induced with intravenous propofol 200 mg, lidocaine 100 mg, and succinylcholine 200 mg, and she was endotracheally intubated. A nerve stimulator–guided lumbar plexus block and an ultrasound-guided popliteal block were placed on the operative limb postintubation and prior to surgical incision. General anesthesia was maintained with inhaled sevoflurane in oxygen/air with expired sevoflurane ranging from 2.1% to 2.5%. An electroencephalogram (EEG) bispectral index was not utilized during the procedure. The surgery proceeded uneventfully for the first 4 hours of operative time without narcotic administration. There were two periods of tourniquet inflation. Sequential compression device placement to the nonoperative limb was not documented during the procedure.

Following the second tourniquet deflation after completion of the femur osteotomy, there was a sudden and sustained decrease in oxygen saturation (nadir SpO_2_ 60%) and EtCO_2_ (10–15 mmHg) with concurrent tachycardia (120–140 bpm). The patient was placed on 100% oxygen, the inhaled sevoflurane was discontinued, and she was hand ventilated without hemodynamic normalization. The expired sevoflurane concentration abruptly decreased alongside the EtCO_2_, 2% to 0.2% over 4 min and remained negligible for the remainder of the case. The endotracheal tube was suctioned with return of clear secretions. Flumazenil was administered in the setting of continued unresponsiveness despite negligible expired sevoflurane. An arterial blood gas showed a metabolic acidosis, elevated lactate, low PaO_2_ (84 mmHg) in setting of 100% FiO_2_, and a large discrepancy between EtCO_2_ (14 mmHg) and PaCO_2_ (51 mmHg). She remained with SpO_2_ 60%–93% for approximately 30 min. A phenylephrine infusion was initiated for hypotension management. Pediatric cardiology was consulted, and a transesophageal echocardiogram demonstrated moderately decreased right ventricular function and a mildly dilated right ventricle. There was no noted intracardiac thrombus, vegetation, or right to left shunting heart lesions. To further aid in diagnosis and treatment, a CT-head and CT-chest were obtained. The CT-head demonstrated concern for possible brainstem infarct versus artifact. The CT-chest demonstrated branching hypoattenuation along the pulmonary arteries in the perihilar region; however, the images were suboptimal due to body habitus and positioning, and it was unclear if these represented a true filling defect or artifact.

**Figure 1:**
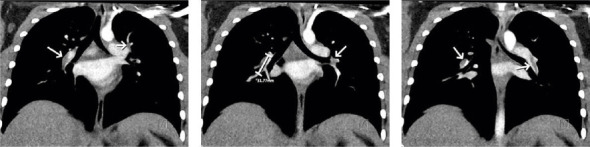
Images from CT-chest on POD3 demonstrating partially occlusive filling defects consistent with pulmonary embolism.

The patient was transported to the pediatric intensive care unit (PICU), and at the time of the patient care transfer, she began to open her eyes and follow commands. Approximately 3 hours had elapsed between sevoflurane discontinuation and beginning of awakening during which only 300 mg of intravenous propofol was given in divided doses due to coughing on the endotracheal tube. She was extubated shortly after arrival in the PICU and prophylactic enoxaparin 40 mg twice daily was initiated given no apparent thrombus at this time and risk of bleeding post major orthopedic surgery. On Postoperative Day (POD) 3, additional imaging was obtained. A lower extremity ultrasound with doppler was performed and was negative for DVT. A repeat CT-chest demonstrated bilateral partially occlusive filling defects within the right lower lobe, subsegmental right middle lobe, subsegmental right upper lobe arteries, and within the left lower and upper lobe subsegmental arteries (Figure 1). Straightening of the interventricular septum was also noted. The PE treatment was initiated with rivaroxaban 20 mg daily. She was subsequently discharged on POD4. After being briefly lost to follow-up, she was admitted approximately 5 months later for anemia during which a repeat CT-chest was obtained and demonstrated complete resolution of the PE.

## 3. Discussion

The change in V/Q ratios caused by PE resulted from relocation of an embolus to the pulmonary vasculature causing obstruction and subsequent redistribution of perfusion to nonoccluded vessels [2]. When there is high V/Q mismatch from significant overperfusion of low-ventilated areas, there is resultant hypoxemia, hypercapnia, and impaired elimination of carbon dioxide. The resultant flow obstruction results in right-sided heart strain and hypotension due to the sudden increased flow seen to the right heart [2]. The hemodynamic changes, blood gas values, and imaging findings seen in our patient are consistent with the diagnosis of PE. In our patient, the calculated P/F ratio on first arterial blood gas was 90, progressed to 101 at time of second blood gas, and was 92 at the time of final blood gas just prior to transport to the PICU, indicating a significant period of hypoxemia. In addition, the PaCO_2_ was elevated on arterial blood gas (51, 54, and 40 mmHg) during which the EtCO_2_ ranged between 13 and 25 mmHg, indicating a significant period of hypercapnia. Other potential etiologies of V/Q mismatch were not seen. Pulmonary vascular or interstitial lung diseases would have resulted in persistent mismatch throughout the case. A low-flow cardiac output state would have been precipitated by an acute change (i.e., an arrhythmia) [3].

It is likely that either venostasis during tourniquet inflation resulted in thrombus formation, or fat embolism related to surgery, and subsequent dislodgement immediately following tourniquet release. In one study of transesophageal echocardiography during orthopedic surgeries showed the echogenic material traveling through the heart in 87% of procedures and major emboli greater than 1 cm were found in 43% [4]. Initially, it was presumed that fat emboli due to the known association with orthopedic procedures involve manipulation of long bones [5, 6]. Fat embolism syndrome (FES) is a systemic disease and is suggested based on diagnostic critieria from Gurd, Lindeque, or Schonfeld [6, 7]. Unfortunately, there is no established FES diagnostic criteria consensus, well-understood mechanism of injury, or treatment strategies [6, 7]. FES is a diagnosis of exclusion and incredibly rare in pediatric patients, though fat emboli have been identified in 30% of pediatric cadavers at autopsy [7]. While multiple mechanisms (including biochemical and mechanical) have been hypothesized to result in FES, the overall general propositions include the response to embolized fat globules, the inflammatory response, and resultant vascular obstruction [6, 7]. FES typically presents with respiratory distress, neurologic impairment, and petechial rash with an insidious onset [5]. Typically, the onset of FES occurs with pulmonary symptoms as presenting feature approximately 24 to 72 h postinjury, though rarely seen as early as 12 h postinjury [7]. The presented patient exhibited some symptoms of FES but did not have an apparent associated rash and presented with cardiopulmonary symptoms while in the operating room. Moreover, bilateral acute femur fractures has the highest incidence of FES [6], whereas our patient had unilateral osteotomies for leg-length discrepancy correction. Imaging, including magnetic resonance imaging, chest radiography, and high-resolution CT may have specific suggestive findings of fat emboli [6, 7]. If FES is suggested, a more thorough workup including ophthalmology evaluation can be considered but was deferred in our case as the main insult resulted in purely pulmonary complications and not complete FES.

There is a vast list of risk factors for pediatric DVT including but not limited to surgery (especially greater than 4 h duration), trauma, increased pediatric age, race, immobility, inherited thrombophilias, oral contraceptive (OCP) use, renal comorbidities, infection, and positive family history [8–11]. In support of thromboembolism, our patient possessed several prothrombotic risk factors including morbid obesity, being of Black race, older pediatric age, orthopedic surgical procedure of long duration, and OCP usage. While not a widely recognized risk for DVT, asthma has been identified as an increased risk factor for PE development [12]. However, this risk is largely seen in patients with recent, chronic oral steroid use to which this cannot be attributed in our patient. At the time of the event, the patient was found to have elevated D-dimer which then normalized on subsequent analysis 18 months later. While inherited thrombophilias such as Factor V Leiden can also increase risk of DVT formation, this patient was not worked up for these disorders. In addition, following informal discussion with pediatric radiologists at our institution, it was deemed more likely thromboembolism than fat embolism given the “rope-like” appearance of the filling defects seen on the repeat CT-chest.

DVT is overall uncommon in children, and the rate of occurrence is approximately 10 times less than in adults [9]. The highest incidence is bimodal, occurring before 1 year of age and increases at 13 years until the risk is similar to adults by age 16 [8, 9]. The most common etiology in the pediatric population is the association with central venous catheters [8, 11]. Adequate fluid balance and early mobilization are mainstays of thromboprophylaxis, and medications can be considered based on each child's risk factors. Scoring of these factors have not been clinically studied, and no guidelines based on specific surgeries exist. Estrogen, obesity (> 95th percentile for weight), and combined OCP increase DVT risk the most [9]. When considering surgical technique and timing, the literature is variable and often contradictory so no recommendations can be made at this time. Discontinuation of OCP in the months prior can be discussed with the patient and family, but a social risk assessment should be weighed in decision-making as well.

Our patient remained unresponsive for multiple hours despite the discontinuation of inhaled anesthetic, as well as the reversal of potentially sedating medications. The differential diagnosis of delayed emergence includes residual effects of medications or impaired metabolism preventing elimination, polypharmacy, metabolic derangements such as acidosis or hypo/hyperglycemia, neurologic insults such as stroke or intracranial hemorrhage, or hypothermia [13]. Laboratory evaluation was not significant for any renal or hepatic impairment or electrolyte abnormalities. Pseudocholinesterase deficiency with residual neuromuscular blockade was briefly considered. However, the patient was breathing spontaneously, would occasionally withdraw limbs to pain, and was tachycardic and hypotensive requiring phenylephrine infusion as opposed to hypertensive. Given decreased responsiveness in the setting of presumed PE, there was concern for possible embolic event resulting in cerebrovascular insult. However, the CT-head was inconclusive. In addition, the patient did not display any neurologic deficits after awakening in the PICU and endotracheal extubation.

Sevoflurane is primarily eliminated via exhalation and is influenced by cardiac output, alveolar ventilation, and solubility [14]. Sevoflurane has a relatively low blood solubility, typically allowing for rapid induction and emergence [14]. However, with prolonged exposure, sevoflurane can redistribute to peripheral tissues and can prolong emergence [14]. In our patient, the long anesthetic duration likely led to a higher peripheral redistribution. While prolonged exposure to inhaled sevoflurane in a morbidly obese patient can lead to delayed emergence, the presented patient had a sudden decrease in expired sevoflurane concentration at the same time as a sudden decrease in EtCO_2_ and associated hypotension and tachycardia. There were occasional low readings of expired sevoflurane concentration but largely remained zero following the embolic event. Therefore, the multiple and scattered bilateral pulmonary emboli likely prevented adequate perfusion and ventilation, interrupted normal sevoflurane elimination despite increased fresh gas flow, and led to a delayed emergence.

## 4. Conclusion

In the case presented, a pediatric patient undergoing long bone osteotomies and fixation developed hemodynamic instability secondary to pulmonary emboli, most likely from acute thromboembolus formation and dislodgement. While the etiology of the embolic event is not definitive, we suspect the PE led to a profound V/Q mismatch hindering gas exchange and the elimination of sevoflurane leading to a profoundly delayed emergence.

## Data Availability

Data sharing is not applicable to this article as no new data were created or analyzed in this study.

## Supporting Information

Supporting Document 1: CARE—Case Report Guidelines and Checklist of Information.
