# Supplementary material for: Profoundly Delayed Emergence due to Pulmonary Embolism
Source: Case Rep Anesthesiol. 2025 Feb 18;2025:6691273. doi: 10.1155/cria/6691273 (PMC11858700; doi:10.1155/cria/6691273)
Supplement: Supporting Information — Additional supporting information can be found online in the Supporting Information section. [file 6691273.f1.pdf]

CARE
case report guidelines

CARE Checklist of information to include when writing a case report

(cc) BY-NC-ND

☑

Topic	Item	Checklist item description	Reported on Line
Title	1	The diagnosis or intervention of primary focus followed by the words "case report"	P1, L1
Key Words	2	2 to 5 key words that identify diagnoses or interventions in this case report, including "case report"	P2, L12-16
Abstract (no references)	3a	Introduction: What is unique about this case and what does it add to the scientific literature?	P2, L8-9
	3b	Main symptoms and/or important clinical findings	P2, L5-8
	3c	The main diagnoses, therapeutic interventions, and outcomes	P2, L6-8
	3d	Conclusion—What is the main "take-away" lesson(s) from this case?	P2, L8-9
Introduction	4	One or two paragraphs summarizing why this case is unique (**may include references**)	P3, L3-8
Patient Information	5a	De-identified patient specific information.	P3, L12-14
	5b	Primary concerns and symptoms of the patient.	P3, L24-42
	5c	Medical, family, and psycho-social history including relevant genetic information	P3, L12-14
	5d	Relevant past interventions with outcomes	N/A
Clinical Findings	6	Describe significant physical examination (PE) and important clinical findings.	P3, L24-42
Timeline	7	Historical and current information from this episode of care organized as a timeline	✓
Diagnostic Assessment	8a	Diagnostic testing (such as PE, laboratory testing, imaging, surveys).	P3, L31-42; P4, L4-12
	8b	Diagnostic challenges (such as access to testing, financial, or cultural)	N/A
	8c	Diagnosis (including other diagnoses considered)	P4, L16-41
	8d	Prognosis (such as staging in oncology) where applicable	N/A
Therapeutic Intervention	9a	Types of therapeutic intervention (such as pharmacologic, surgical, preventive, self-care)	P4, L2-12
	9b	Administration of therapeutic intervention (such as dosage, strength, duration)	P4, L9
	9c	Changes in therapeutic intervention (with rationale)	P4, L2-12
Follow-up and Outcomes	10a	Clinician and patient-assessed outcomes (if available)	P4, L10-12
	10b	Important follow-up diagnostic and other test results	P4, L10-12
	10c	Intervention adherence and tolerability (How was this assessed?)	P4, L2-12
	10d	Adverse and unanticipated events	P3, L24-42
Discussion	11a	A scientific discussion of the strengths AND limitations associated with this case report	P4, L16-41; P5, L6-41
	11b	Discussion of the relevant medical literature **with references**.	Discussion section
	11c	The scientific rationale for any conclusions (including assessment of possible causes)	P5, L16-28
	11d	The primary "take-away" lessons of this case report (without references) in a one paragraph conclusion	Conclusion section
Patient Perspective	12	The patient should share their perspective in one to two paragraphs on the treatment(s) they received	N/A
Informed Consent	13	Did the patient give informed consent? Please provide if requested	Yes ☒ No ☐
